# Different outer membrane *c*‐type cytochromes are involved in direct interspecies electron transfer to *Geobacter* or *Methanosarcina* species

**DOI:** 10.1002/mlf2.12037

**Published:** 2022-09-23

**Authors:** Dawn E. Holmes, Jinjie Zhou, Jessica A. Smith, Caiqin Wang, Xinying Liu, Derek R. Lovley

**Affiliations:** ^1^ Department of Microbiology University of Massachusetts‐Amherst Amherst Massachusetts USA; ^2^ Department of Physical and Biological Science Western New England University Springfield Massachusetts USA; ^3^ Institute for Advanced Study Shenzhen University Shenzhen China; ^4^ Department of Biomolecular Sciences Central Connecticut State University New Britain Connecticut USA; ^5^ College of Environment Zhejiang University of Technology Hangzhou China; ^6^ College of Environmental Science and Engineering Beijing Forestry University Beijing China

**Keywords:** *c*‐type cytochrome, direct interspecies electron transfer (DIET), extracellular electron transfer, *Geobacter*, *Methanosarcina*

## Abstract

Direct interspecies electron transfer (DIET) may be most important in methanogenic environments, but mechanistic studies of DIET to date have primarily focused on cocultures in which fumarate was the terminal electron acceptor. To better understand DIET with methanogens, the transcriptome of *Geobacter metallireducens* during DIET‐based growth with *G. sulfurreducens* reducing fumarate was compared with *G. metallireducens* grown in coculture with diverse *Methanosarcina*. The transcriptome of *G. metallireducens* cocultured with *G. sulfurreducens* was significantly different from those with *Methanosarcina*. Furthermore, the transcriptome of *G. metallireducens* grown with *Methanosarcina barkeri*, which lacks outer‐surface *c*‐type cytochromes, differed from those of *G. metallireducens* cocultured with *M. acetivorans* or *M. subterranea*, which have an outer‐surface *c*‐type cytochrome that serves as an electrical connect for DIET. Differences in *G. metallireducens* expression patterns for genes involved in extracellular electron transfer were particularly notable. Cocultures with *c*‐type cytochrome deletion mutant strains, ∆Gmet_0930, ∆Gmet_0557 and ∆Gmet_2896, never became established with *G. sulfurreducens* but adapted to grow with all three *Methanosarcina*. Two porin–cytochrome complexes, PccF and PccG, were important for DIET; however, PccG was more important for growth with *Methanosarcina*. Unlike cocultures with *G. sulfurreducens* and *M. acetivorans*, electrically conductive pili were not needed for growth with *M. barkeri*. *Shewanella oneidensis*, another electroactive microbe with abundant outer‐surface *c*‐type cytochromes, did not grow via DIET. The results demonstrate that the presence of outer‐surface *c*‐type cytochromes does not necessarily confer the capacity for DIET and emphasize the impact of the electron‐accepting partner on the physiology of the electron‐donating DIET partner.

## Impact statement

Direct interspecies electron transfer (DIET) is significant in methane‐generating soils and sediments that contribute to atmospheric methane, as well as in anaerobic digesters converting organic wastes to methane that can be recovered as fuel. *Geobacter* species are important electron‐donating partners for DIET in these environments. The finding that the route for extracellular electron transfer from *G. metallireducens* during DIET depends on the physiological characteristics of the electron‐accepting partner demonstrates the need to investigate DIET electron transfer pathways with a diversity of environmentally relevant methanogens to better understand and potentially manipulate the DIET process.

## INTRODUCTION

Direct interspecies electron transfer (DIET) is proposed to play a major role in the global cycling of methane, an important greenhouse gas, and in the generation of methane biofuel from organic wastes. Syntrophic communities producing or consuming methane in anaerobic soils and sediments share electrons via DIET^1^, ^2^. Promoting DIET in anaerobic digesters through changes in digester design or with the addition of electrically conductive materials can accelerate anaerobic digestion^3^, ^4^. Thus, understanding the mechanisms for electron exchange during DIET could contribute to better modeling of carbon and electron flux in natural anaerobic environments and could suggest better strategies for enhancing DIET during organic waste treatment.

Until now, studies on the routes for interspecies electron transfer during DIET have primarily focused on the mechanisms of electron uptake by the electron‐accepting partner^5^, ^6^, ^7^, ^8^, ^9^. These studies have demonstrated that the electrical connects on the outer cell surface of electron‐accepting partners can be dramatically different. For example, *Geobacter sulfurreducens* displays a network of electrically conductive pili (e‐pili) and outer‐surface multiheme *c*‐type cytochromes to facilitate electron uptake during DIET^5^, ^6^, ^10^. In contrast, some methanogens can express electrically conductive archaella^11^, but archaella are not known to participate in DIET and do not form a multifiber conductive extracellular mesh, like *Geobacter* e‐pili. Furthermore, some methanogens, such as *Methanothrix harundinacea, Methanobacterium electrotrophus* strain YSL and *Methanosarcina barkeri*, are capable of DIET despite a lack of outer‐surface *c*‐type cytochromes^7^, ^8^, ^12^, ^13^, ^14^, ^15^, ^16^. The electrical contacts for DIET in these methanogens are unknown. However, another methanogen, *M. acetivorans*, expresses an outer‐surface multiheme *c*‐type cytochrome, MmcA, which is a key electrical contact for DIET^9^.

This major difference in mechanisms for DIET‐based electron uptake between *M. barkeri* and *M. acetivorans* is just one of the physiological dissimilarities between two clades of *Methanosarcina* species. Type I *Methanosarcina* species, like *M. barkeri*, predominate in waste digesters and other high‐energy environments and have different mechanisms for energy conservation than Type II *Methanosarcina*, such as *M. acetivorans*, which are better adapted for growth in organic‐poor soils and sediments^15^. It might be expected that differences in the diversity of electrical contacts on electron‐accepting partners for DIET could influence the routes for electron transfer from electron‐donating partners.

Furthermore, the majority of studies on DIET mechanisms have focused on cocultures in which fumarate‐reducing *G. sulfurreducens* was the electron‐accepting partner. DIET to a fumarate‐reducing partner is unlikely to be important in anaerobic environments because fumarate is not a commonly abundant electron acceptor. In addition, most simple organic substrates can be directly metabolized within individual fumarate‐respiring microbes, which means that syntrophy is not required. In contrast, the inability of most methanogens to use multicarbon electron donors other than acetate necessitates syntrophic metabolism with interspecies electron transfer. The electron carriers that provide electrons to support fumarate reduction during DIET could be much different than those required to promote the reduction of carbon dioxide to methane because the mid‐point potential of the carbon dioxide/methane redox couple is much more negative than the fumarate/succinate couple.

A diversity of electron‐donating partners for DIET have been identified, but in most instances, genetically tractable isolates for mechanistic studies are not available. For example, pure cultures are not available for the archaea proposed to function as electron‐donating partners for DIET in consortia catalyzing anaerobic methane oxidation coupled to sulfate reduction^17^, ^18^, ^19^. Tools for genetic manipulation have not been developed for bacteria, such as *Syntrophus aciditrophicus* ^20^, *Rhodoferax ferrireducens* ^8^ and *Desulfovibrio* strain JY^21^, shown to serve as the electron‐donating partner for DIET in defined cocultures.

Therefore, *G. metallireducens*, which is genetically tractable^22^, has served as the primary pure culture model for an electron‐donating partner in DIET. *G. metallireducens* is an attractive model for DIET studies because of its environmental relevance. *Geobacter* species are important electron‐donating DIET partners in some anaerobic waste digesters^4^, ^23^ and in rice paddy soils, an important source of atmospheric methane^24^. *G. metallireducens* is a good candidate for studying DIET in defined systems because it can oxidize ethanol and other short‐chain alcohols with electron transfer to extracellular electron acceptors without generating H_2_ or formate^5^, ^6^, ^10^, ^25^, ^26^.

Here, we report that DIET transcriptomes suggest that the physiology of the electron‐accepting partner directly impacts the physiology of *G. metallireducens* serving as the electron‐donating partner. Gene deletion studies suggested different routes for electron transport, depending on which partner *G. metallireducens* was paired with. We also demonstrate that in many instances, *G. metallireducens*/*Methanosarcina* cocultures adapted over time to gene deletions that initially disrupted DIET. Attempts to simplify the study of DIET extracellular electron exchange by initiating DIET cocultures with *Shewanella oneidensis*, an electroactive microbe with a less complicated array of outer‐surface electron transport options, were unsuccessful.

## RESULTS AND DISCUSSION

### Gene expression patterns suggest that the electron‐accepting partner influences routes for DIET

**Figure 1:**
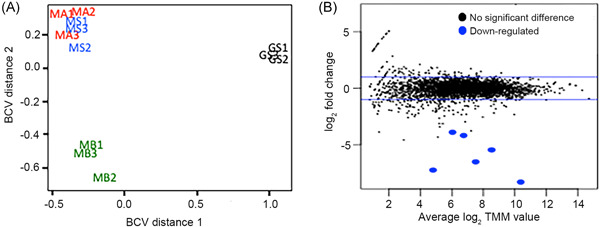
Biological coefficient of variation (BCV) and mean‐difference (MD) plots comparing various RNAseq libraries. (A) Comparison of *G. metallireducens* RNAseq libraries from cocultures with *Methanosarcina barkeri* (MB), *M. acetivorans* (MA), *M. subterranea* (MS) and *Geobacter sulfurreducens* (GS) using multidimensional scaling analysis with BCV method. (B) MD plots generated with plotMD from the LIMMA package in RStudio comparing *G. metallireducens* normalized transcripts from cocultures grown with *M. acetivorans* or *M. subterranea* as the electron‐accepting partner. log_2_‐fold differences in expression for each gene (log_2_‐fold change) are plotted on the *y*‐axis and the log of normalized trimmed means of M (TMM) values for each gene is plotted on the *x*‐axis. Each dot represents a different gene and any dot that falls between the blue lines represents a gene that is not differentially expressed (less than one‐fold up or down). If the differential expression is not statistically significant, the gene is represented by a black dot. The blue dots represent genes that were significantly down‐regulated (*p* < 0.05) in *M*. *subterranea* compared to *M*. *acetivorans*. All libraries were done in triplicate and were normalized with the TMM method.

DIET between *G. metallireducens* and *G. sulfurreducens* was compared to DIET between *G. metallireducens* and three *Methanosarcina* species: *M. barkeri* (MB), a Type I *Methanosarcina* that lacks outer‐surface *c*‐type cytochromes^15^ and *M. acetivorans* (MA) and *M. subterranea* (MS), Type II *Methanosarcina* with the outer‐surface multiheme *c*‐type cytochrome MmcA that serves an essential electrical contact for DIET^9^. Multidimensional scaling analysis generated with the biological coefficient of variation (BCV) method and mean difference plots (MD plots) revealed that the transcriptomes of *G. metallireducens* grown in DIET‐based coculture with *Methanosarcina* species were significantly different from those of *G. metallireducens* grown in coculture with *G. sulfurreducens* (Figures 1 and S1, Tables S1–, S3). Furthermore, the transcriptomes of *G. metallireducens* grown with the Type I *Methanosarcina*, *M. barkeri*, were significantly different from those of *G. metallireducens* grown with the Type II *Methanosarcina* species, *M. acetivorans* and *M. subterranea*. Both MDS (Figure 1A) and MD plots (Figure 1B) revealed that the transcriptomes of *G. metallireducens* grown with either of the Type II *Methanosarcina* were similar. These results indicate that the properties of the electron‐accepting DIET partner substantially influence the physiological status of *G. metallireducens* during growth via DIET. Differences in gene expression suggested substantial differences in many aspects of *G. metallireducens* physiology (Figure S2). Here, we focus on the expression of possible outer‐surface electrical connects for DIET.

**Figure 2:**
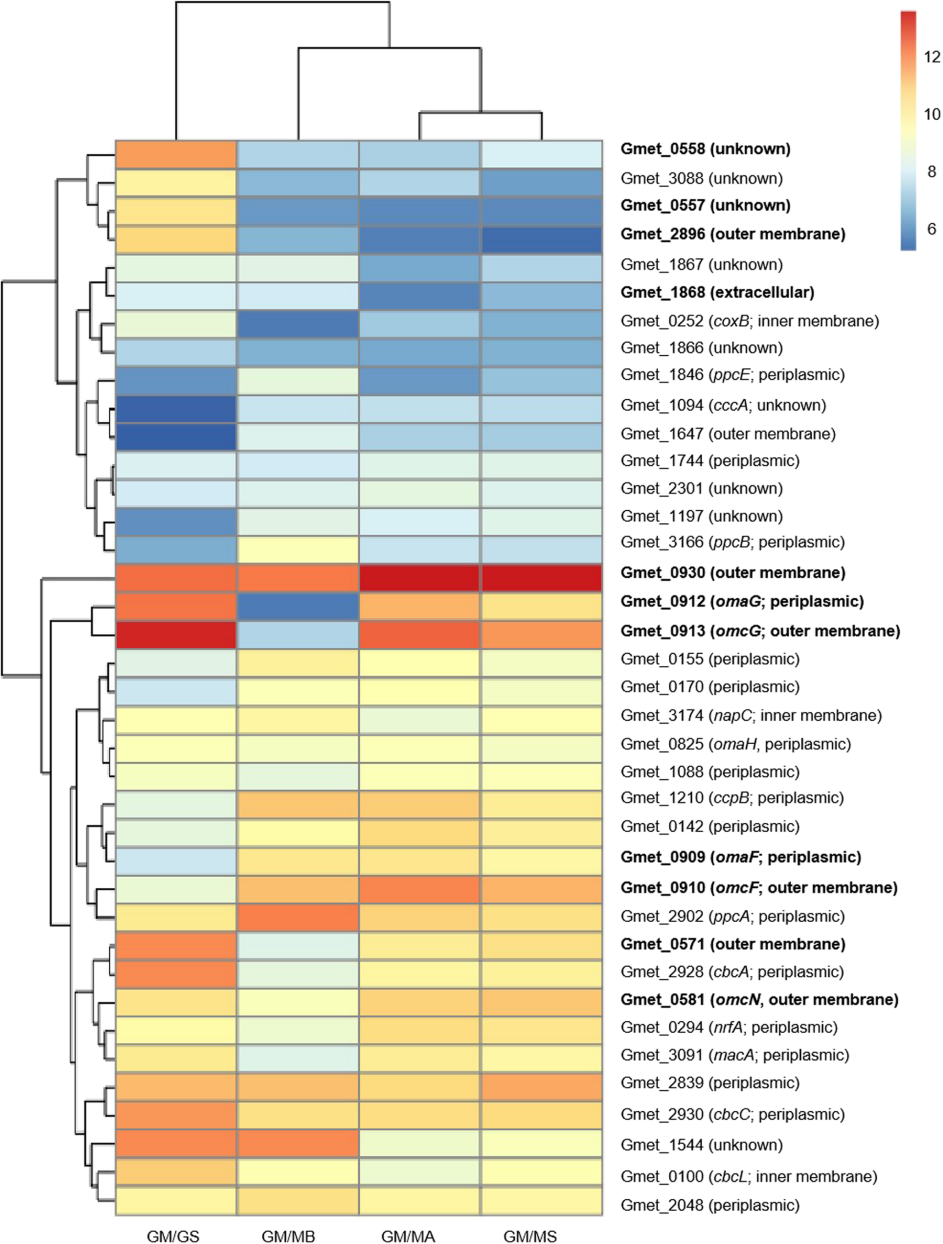
Heatmap showing differences in cytochrome mRNA transcripts. log_2_ read values normalized with the TMM method for *Geobacter metallireducens c*‐type cytochrome genes from four different RNAseq coculture libraries (GM/GS, *G. metallireducens* and *G. sulfurreducens*; GM/MB, *G. metallireducens* and *Methanosarcina barkeri*; GM/MA, *G. metallireducens* and *M. acetivorans*; GM/MS, *G. metallireducens* and *M. subterranea*) that had log_2_ values above the median TMM values. mRNA, messenger RNA; TMM, trimmed means of M values. Highlighted genes displayed phenotypes when grown in co‐cultures.

Some of the most significant differences in *G. metallireducens* gene expression patterns during growth with the different types of electron‐accepting partners were for genes coding for multiheme *c*‐type cytochromes (Figure 2, Tables 1, 2, 3, S1 and S2). The pattern of cytochrome gene expression was most similar in *G. metallireducens* grown with the two Type II *Methanosarcina* and the expression patterns between *G. metallireducens* and the Type I and Type II *Methanosarcina* were more similar to each other than to *G. metallireducens* grown with *G. sulfurreducens* as the electron‐accepting partner. Therefore, similarities and differences in *G. metallireducens* cytochrome gene expression and the impact of targeted gene deletions on the ability of *G. metallireducens* to establish DIET with different electron‐accepting partners were further evaluated.

**Table 1:** Differences in expression of genes coding for electron transfer proteins in *Geobacter metallireducens* that were important for DIET when *G. sulfurreducens* was the electron‐accepting partner.

Locus ID	Annotation	Gene	Location	GS vs. MB	GS vs. MA	GS vs. MS
Gmet_0558	*c*‐type cytochrome protein, 27 hemes		Unknown	18.28	19.68	13.50
Gmet_0557	*c*‐type cytochrome protein, 4 hemes		Unknown	18.02	22.42	24.19
Gmet_2896	*c*‐type cytochrome protein, 4 hemes		Outer membrane/surface	17.01	33.12	46.11
Gmet_0930	*c*‐type cytochrome, 8 hemes		Outer membrane/surface	NS	−2.80	−2.57
Gmet_1868	c‐type cytochrome, 4 hemes		Outer membrane/surface	NS	4.12	2.31
Gmet_0571	*c*‐type cytochrome protein, 34 hemes		Outer membrane/surface	12.63	3.29	2.72
Gmet_0581	*c*‐type cytochrome protein, 34 hemes	*omcN*	Outer membrane/surface	2.31	NS	NS
Gmet_0911	Porin protein from PccG complex	*ombG*	Outer membrane/surface	80.67	NS	4.0
Gmet_0912	8 heme periplasmic *c*‐type cytochrome from PccG complex	*omaG*	Periplasmic	96.91	2.01	3.58
Gmet_0913	9 heme outer membrane *c*‐type cytochrome from PccG	*omcG*	Outer membrane/surface	70.53	2.34	3.43
Gmet_0908	Porin protein from PccF complex	*ombF*	Outer membrane/surface	−3.57	−4.76	−2.41
Gmet_0909	9 heme periplasmic *c*‐type cytochrome from PccF complex	*omaF*	Periplasmic	−6.50	−7.08	−3.81
Gmet_0910	10 heme *c*‐type cytochrome from PccF complex	*omcF*	Outer membrane/surface	−6.41	−11.42	−6.30
Gmet_1399	Type IV major pilin subunit, PilA	*pilA*	Outer membrane/surface	−4.31	1.64	1.76
Gmet_1400	Short pilin chaperone protein, Spc	*spc*	Outer membrane/surface	−7.85	NS	NS
Gmet_2163	Multicopper oxidase protein	*ompB*	Outer membrane/surface	3.89	7.66	10.72
Gmet_2928	7 heme *c*‐type cytochrome protein from CbcABCDE	*cbcA*	Periplasmic	11.09	4.34	4.13
Gmet_2929	*b*‐type cytochrome from CbcABCDE complex	*cbcB*	Inner membrane	7.85	6.08	5.22
Gmet_2930	11 heme *c*‐type cytochrome protein, CbcABCD complex	*cbcC*	Periplasmic	2.16	2.0	2.14
Gmet_2931	Monoheme *c*‐type cytochrome protein from CbcABCDE complex	*cbcD*	Periplasmic	3.18	2.21	2.50
Gmet_2932	Membrane protein from CbcABCDE complex	*cbcE*	Inner membrane	4.46	1.74	1.80
Gmet_0252	Monoheme *c*‐type cytochrome protein	*coxB*	Inner membrane	6.65	2.46	4.09
Gmet_0325	4 heme *c*‐type cytochrome protein from CbcSTU‐2 complex	*cbcS‐2*	Periplasmic	5.37	11.80	3.18
Gmet_0327	*b*‐type cytochrome from CbcSTU‐1 complex	*cbcU‐2*	Inner membrane	2.25	9.48	4.64
Gmet_3519	4Fe‐4S ferredoxin from CbcSTU‐1 complex	*cbcT‐1*	Periplasmic	NS	3.47	3.44
Gmet_3520	*b*‐type cytochrome from CbcSTU‐2 complex	*cbcU‐1*	Inner membrane	3.86	9.98	4.14
Gmet_0100	Quinone oxidoreductase cytochrome, 9 hemes	*cbcL*	Inner membrane	2.96	4.51	3.37
Gmet_3091	*c*‐type cytochrome protein, 2 hemes	*macA*	Periplasmic	3.82	NS	NS
Gmet_3165	*c*‐type cytochrome protein, 3 hemes	*ppcC*	Periplasmic	3.31	2.29	NS
Gmet_0580	*c*‐type cytochrome protein, 21 hemes		Outer membrane/surface	3.02	NS	NS
Gmet_0600	*c*‐type cytochrome protein, 19 hemes		Outer membrane/surface	2.21	NS	NS
Gmet_0575	*c*‐type cytochrome protein, 27 hemes		Outer membrane/surface	2.01	3.03	2.86
Gmet_3088	*c*‐type cytochrome protein, 8 hemes		Unknown	8.84	5.59	12.98
Gmet_2898	*c*‐type cytochrome protein, 8 hemes		Unknown	6.81	NS	NS
Gmet_2470	*c*‐type cytochrome protein, 34 hemes		Unknown	5.67	2.05	1.70
Gmet_2899	*c*‐type cytochrome protein, 9 hemes		Unknown	5.43	NS	NS
Gmet_1087	*c*‐type cytochrome protein, 1 heme		Unknown	4.46	8.40	6.56
Gmet_0601	*c*‐type cytochrome protein, 8 hemes		Unknown	2.34	NS	NS
Gmet_0534	*c*‐type cytochrome protein, 5 hemes	*cbcR*	Periplasmic	NS	3.36	3.10
Gmet_0121	*c*‐type cytochrome protein, 2 hemes		Unknown	NS	2.12	2.09

Values represent fold difference between cocultures grown with *G. sulfurreducens* (GS) compared to cocultures grown with the three different *Methanosarcina* species (*M. barkeri* [MB]; *M. acetivorans* [MA]; *M. subterranea* [MS]). Negative values show that the gene was more highly expressed by *G. metallireducens* grown in coculture with the methanogen. *p* values are available in Table S2 but all comparisons shown have *p* values that are <0.05. NS, no significant difference.

**Table 2:** Genes coding for electron transport proteins in *Geobacter metallireducens* that had greater than two‐fold differences in expression in cocultures grown with *Methanosarcina barkeri* compared to cocultures grown with *G. sulfurreducens* (GS) and the Type II *Methanosarcina* (*M. acetivorans* [MA] and *M. subterranea* [MS]).

Locus ID	Annotation	Gene	Location	MB vs. GS	MB vs. MA	MB vs. MS
Gmet_2119	Cytochrome *b* from PcmABCDEFG complex	*pcmC*	Inner membrane	104.99	9.93	NS
Gmet_2117	Membrane protein from PcmABCDEFG complex	*pcmA*	Inner membrane	10.83	9.17	7.19
Gmet_1019	*c*‐type cytochrome, 2 hemes	*narC*	Inner membrane	8.44	NS	NS
Gmet_0330	Monoheme *c*‐type cytochrome	*narH*	Inner membrane	5.37	NS	NS
Gmet_1809	*c*‐type cytochrome, 5 hemes	*actA*	Inner membrane	5.15	NS	NS
Gmet_1923	*b*‐type cytochrome from CbcWXV complex	*cbcW*	Inner membrane	3.84	1.54	NS
Gmet_1922	Rieske 2Fe‐2S protein from CbcWXV complex	*cbcV*	Inner membrane	1.92	1.64	2.13
Gmet_2120	Cytochrome *b* from PcmABCDEFG complex	*pcmD*	Inner membrane	NS	4.66	NS
Gmet_2122	4 heme *c*‐type cytochrome from PcmABCDEFG complex	*pcmF*	Periplasmic	123.26	6.85	83.58
Gmet_2123	4 heme *c*‐type cytochrome from PcmABCDEFG complex	*pcmG*	Periplasmic	8.94	20.04	47.60
Gmet_3166	*c*‐type cytochrome, 2 hemes	*ppcB*	Periplasmic	7.65	3.13	3.37
Gmet_1210	*c*‐type cytochrome, 2 hemes	*ccpB*	Periplasmic	7.59	NS	2.11
Gmet_2156	*c*‐type cytochrome, 9 hemes		Periplasmic	7.19	2.87	NS
Gmet_1703	*c*‐type cytochrome, 7 hemes		Periplasmic	6.71	7.63	3.59
Gmet_0909	9 heme *c*‐type cytochrome from PccF complex	*omaF*	Periplasmic	6.50	NS	NS
Gmet_1846	*c*‐type cytochrome, 2 hemes	*ppcE*	Periplasmic	6.12	5.52	3.33
Gmet_0328	*c*‐type cytochrome, 2 hemes	*narC*	Periplasmic	5.80	2.62	2.50
Gmet_0335	*c*‐type cytochrome, 3 hemes	*ppcF*	Periplasmic	5.52	2.95	2.64
Gmet_2902	*c*‐type cytochrome, 2 hemes	*ppcA*	Periplasmic	4.31	2.42	3.16
Gmet_1924	5 heme *c*‐type cytochrome from CbcWXV complex	*cbcX*	Periplasmic	4.24	2.98	2.85
Gmet_0155	Monoheme *c*‐type cytochrome		Periplasmic	3.50	NS	NS
Gmet_0170	*c*‐type cytochrome, 10 hemes		Periplasmic	3.12	NS	NS
Gmet_0142	*c*‐type cytochrome, 8 hemes		Periplasmic	2.42	NS	NS
Gmet_0910	10 heme *c*‐type cytochrome from PccF complex	*omcF*	Outer membrane/surface	6.41	NS	NS
Gmet_0908	Porin protein from PccF complex	*ombF*	Outer membrane/surface	3.57	NS	NS
Gmet_1868	*c*‐type cytochrome, 4 hemes		Outer membrane/surface	NS	4.41	2.35
Gmet_2121	Rieske 2Fe‐2S protein from PcmABCDEFG complex	*pcmE*	Unknown	53.94	11.03	NS
Gmet_2118	4Fe‐4S ferredoxin protein from PcmABCDEFG complex	*pcmB*	Unknown	18.16	3.76	NS
Gmet_1647	*c*‐type cytochrome, 2 hemes		Unknown	9.35	NS	NS
Gmet_1094	*c*‐type cytochrome, 2 hemes	*cccA*	Unknown	6.02	NS	NS
Gmet_1197	*c*‐type cytochrome, 5 hemes		Unknown	5.64	NS	NS
Gmet_1191	Monoheme *c*‐type cytochrome		Unknown	4.49	NS	NS
Gmet_1814	Monoheme *c*‐type cytochrome	*actE*	Unknown	4.16	NS	NS
Gmet_3506	*c*‐type cytochrome, 2 hemes		Unknown	3.49	NS	NS
Gmet_2432	Monoheme P460 *c*‐type cytochrome		Unknown	2.71	NS	NS
Gmet_2896	*c*‐type cytochrome, 4 hemes		Unknown	NS	2.01	2.63

*p* values are available in Table S2 but all comparisons shown have *p* values that are <0.05. NS, no significant difference.

**Table 3:** Genes coding for electron transport proteins in *Geobacter metallireducens* that had greater than two‐fold differences in expression in cocultures grown with the Type II *Methanosarcina* (*M. acetivorans* [MA]) than with *G. sulfurreducens* (GS), *M. barkeri* (MB) or *M. subterranea* (MS).

Locus ID	Annotation	Gene	Location	MA vs. GS	MA vs. MB	MA vs. MS
Gmet_0533	Transmembrane protein from CbcMNOPQR complex	*cbcQ*	Inner membrane	6.59	5.36	NS
Gmet_1019	*c*‐type cytochrome, 2 hemes	*narC*	Inner membrane	NS	3.71	NS
Gmet_0252	Monoheme *c*‐type cytochrome	*coxB*	Inner membrane	NS	2.68	NS
Gmet_0539	*b*‐type cytochrome from CbcMNOPQR complex	*cbcP*	Inner membrane	NS	2.28	NS
Gmet_0330	Monoheme *c*‐type cytochrome	*narH*	Inner membrane	4.02	NS	NS
Gmet_0538	Transmembrane protein from CbcMNOPQR complex	*cbcO*	Inner membrane	NS	NS	NS
Gmet_0912	8 heme periplasmic *c*‐type cytochrome from PccG complex	*omaG*	Periplasmic	NS	54.54	NS
Gmet_3091	*c*‐type cytochrome, 2 hemes	*ccpA*	Periplasmic	NS	3.92	NS
Gmet_0294	*c*‐type cytochrome, 4 hemes	*nrfA*	Periplasmic	NS	3.83	NS
Gmet_0232	Monoheme *c*‐type cytochrome		Periplasmic	NS	2.64	NS
Gmet_2928	7 heme *c*‐type cytochrome from CbcABCD complex	*cbcA*	Periplasmic	NS	2.52	NS
Gmet_0537	10 heme *c*‐type cytochrome from CbcMNOPQR complex	*cbcN*	Periplasmic	NS	2.21	NS
Gmet_0142	*c*‐type cytochrome, 8 hemes		Periplasmic	5.15	2.09	NS
Gmet_0155	Monoheme *c*‐type cytochrome		Periplasmic	2.31	NS	NS
Gmet_2122	4 heme *c*‐type cytochrome from PcmABCDEFG complex	*pcmF*	Periplasmic	NS	NS	15.46
Gmet_0909	9 heme periplasmic *c*‐type cytochrome from PccF complex	*omaF*	Periplasmic	7.08	NS	NS
Gmet_0534	5 heme *c*‐type cytochrome from CbcMNOPQR complex	*cbcR*	Periplasmic	NS	NS	NS
Gmet_0911	Porin protein from PccG complex	*ombG*	Outer membrane/surface	NS	41.47	NS
Gmet_0913	9 heme outer membrane *c*‐type cytochrome from PccG complex	*omcG*	Outer membrane/surface	NS	37.16	NS
Gmet_0580	*c*‐type cytochrome, 21 hemes		Outer membrane/surface	NS	4.92	NS
Gmet_0908	Porin protein from PccF complex	*ombF*	Outer membrane/surface	4.76	NS	NS
Gmet_0571	*c*‐type cytochrome, 34 hemes		Outer membrane/surface	NS	3.79	NS
Gmet_0930	*c*‐type cytochrome, 8 hemes	*omcZ*	Outer membrane/surface	2.80	2.75	NS
Gmet_0600	*c*‐type cytochrome, 19 hemes		Outer membrane/surface	NS	2.20	NS
Gmet_0910	10 heme *c*‐type cytochrome from PccF complex	*omcF*	Outer membrane/surface	11.42	NS	NS
Gmet_1647	*c*‐type cytochrome, 2 hemes		Outer membrane/surface	5.03	NS	NS
Gmet_2626	Monoheme P460 *c*‐type cytochrome		Unknown	NS	5.50	NS
Gmet_2899	*c*‐type cytochrome, 9 hemes		Unknown	NS	5.15	NS
Gmet_2898	*c*‐type cytochrome, 8 hemes		Unknown	NS	4.77	NS
Gmet_0581	*c*‐type cytochrome, 34 hemes		Unknown	NS	3.40	NS
Gmet_0679	*c*‐type cytochrome, 5 hemes		Unknown	2.60	3.20	NS
Gmet_2470	*c*‐type cytochrome, 34 hemes		Unknown	NS	2.75	NS
Gmet_0733	Monoheme *c*‐type cytochrome		Unknown	NS	2.41	NS
Gmet_0601	*c*‐type cytochrome, 8 hemes		Unknown	NS	2.34	NS
Gmet_1191	Monoheme *c*‐type cytochrome		Unknown	6.24	NS	NS

*p* values are available in Table S2 but all comparisons shown have *p* values that are <0.05. NS, no significant difference.

### Different outer‐surface *c*‐type cytochromes are required for DIET with *G*. *sulfurreducens* than DIET with *Methanosarcina*

Multiheme, *c*‐type cytochromes positioned on the outer cell surface can serve as a key electrical contact for interspecies electron transfer^27^. Gene expression patterns and the differential impact of deleting genes for outer‐surface *c*‐type cytochromes suggested that the relative importance of individual *G. metallireducens* outer‐surface cytochromes depended upon which microbe served as the electron‐accepting partner for DIET.

For example, a major difference between *G. sulfurreducens* or *Methanosarcina* species serving as the electron‐accepting partner was the apparent role of the multiheme *c*‐type cytochromes Gmet_0558, Gmet_0557 and Gmet_2896. Software (PSORTb version 3.0) designed to predict cellular localization^28^ did not specify a location for these cytochromes. However, the presence of a transmembrane helix in Gmet_0557 suggests that it is membrane‐associated and the Gmet_2896 cytochrome was recovered in outer membrane preparations of *G. metallireducens* ^29^, ^30^. The large number of hemes^27^ predicted for Gmet_0558 and the fact that it has high homology (67% amino acid identity) to OmcO, an outer membrane *c*‐type cytochrome in *G. sulfurreducens* ^31^, also suggests that it may be an outer‐surface electrical contact.

**Figure 3:**
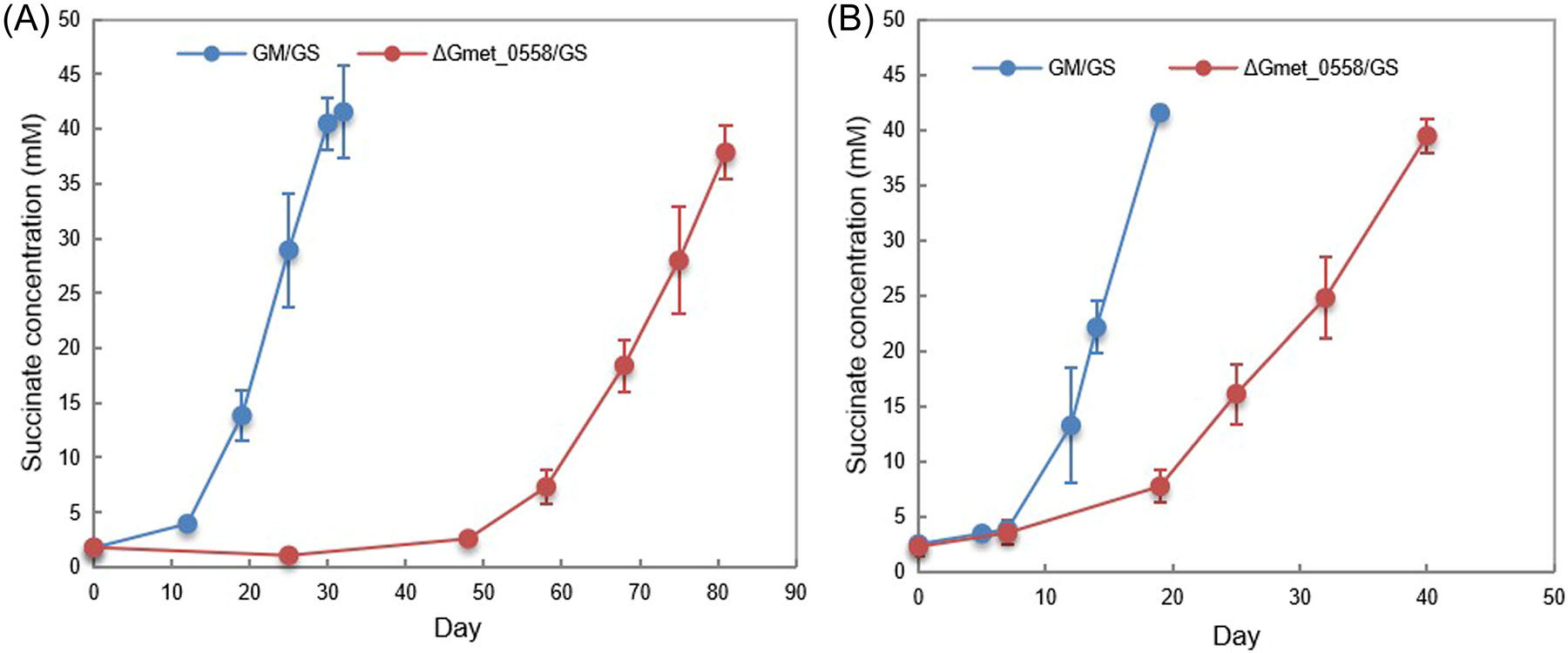
Growth of cocultures with wild‐type *Geobacter metallireducens* or the deletion mutant strain ΔGmet_0558 and *G. sulfurreducens*. Succinate production during (A) the first transfer when aggregates first became established or (B) the fourth transfer after aggregates had been established. Ethanol (10 mM) was provided as the electron donor and fumarate (45 mM) was provided as the electron acceptor. Error bars represent the standard deviation from triplicate cultures.

The genes for all three of these cytochromes were more highly expressed when *G. sulfurreducens* was the electron‐accepting partner than with any of the *Methanosarcina* species (Table 1). Deleting any of these three genes had no impact on establishing cocultures with the three *Methanosarcina* species (Figure S3). However, cocultures initiated with *G. sulfurreducens* and Gmet_0557‐ or Gmet_2896‐deficient strains did not grow and cocultures initiated with a Gmet_0558‐deficient mutant had a more extensive lag period than cocultures initiated with wild‐type *G. metallireducens* (Figure 3A). Even after further adaption with three more transfers, the cocultures with the Gmet_0558‐deficient strain reduced fumarate at rates that were 2.3 times slower (*p* = 1.15 × 10^−5^) than cocultures with wild‐type *G. metallireducens* (Figure 3B). These results suggest that Gmet_0558, Gmet_0557 and Gmet_2896 are all important for extracellular electron transfer when *G. sulfurreducens* is the electron‐accepting partner, but they are not essential for DIET with *Methanosarcina* with or without outer‐surface *c*‐type cytochrome electrical connects. Previous gene‐deletion studies indicated that Gmet_0558, Gmet_0557 and Gmet_2896 are required for growth with insoluble Fe(III) oxide as the electron acceptor^29^. Thus, the concept that *Geobacter* extracellular electron transfer to other microbial species requires the same outer‐surface electron transport components as electron transport to Fe(III) oxide does not appear to hold when *Methanosarcina* species are the partner for DIET.

Gmet_0930 represents another instance in which an outer‐surface *c*‐type cytochrome was essential for DIET to *G. sulfurreducens* and Fe(III) oxide reduction, but not for DIET with *Methanosarcina* species. Gmet_0930 encodes an eight‐heme outer‐surface *c*‐type cytochrome that is homologous to OmcZ (GSU2076) in *G. sulfurreducens*; they have 45% amino acid identity, they both have eight hemes and are predicted to be extracellular and have putative immunoglobulin‐like fold domains. Deletion of the gene for OmcZ had no impact on Fe(III) oxide reduction in *G. sulfurreducens* ^32^, but *G. metallireducens* requires Gmet_0930 to reduce Fe(III) oxide^29^.

**Figure 4:**
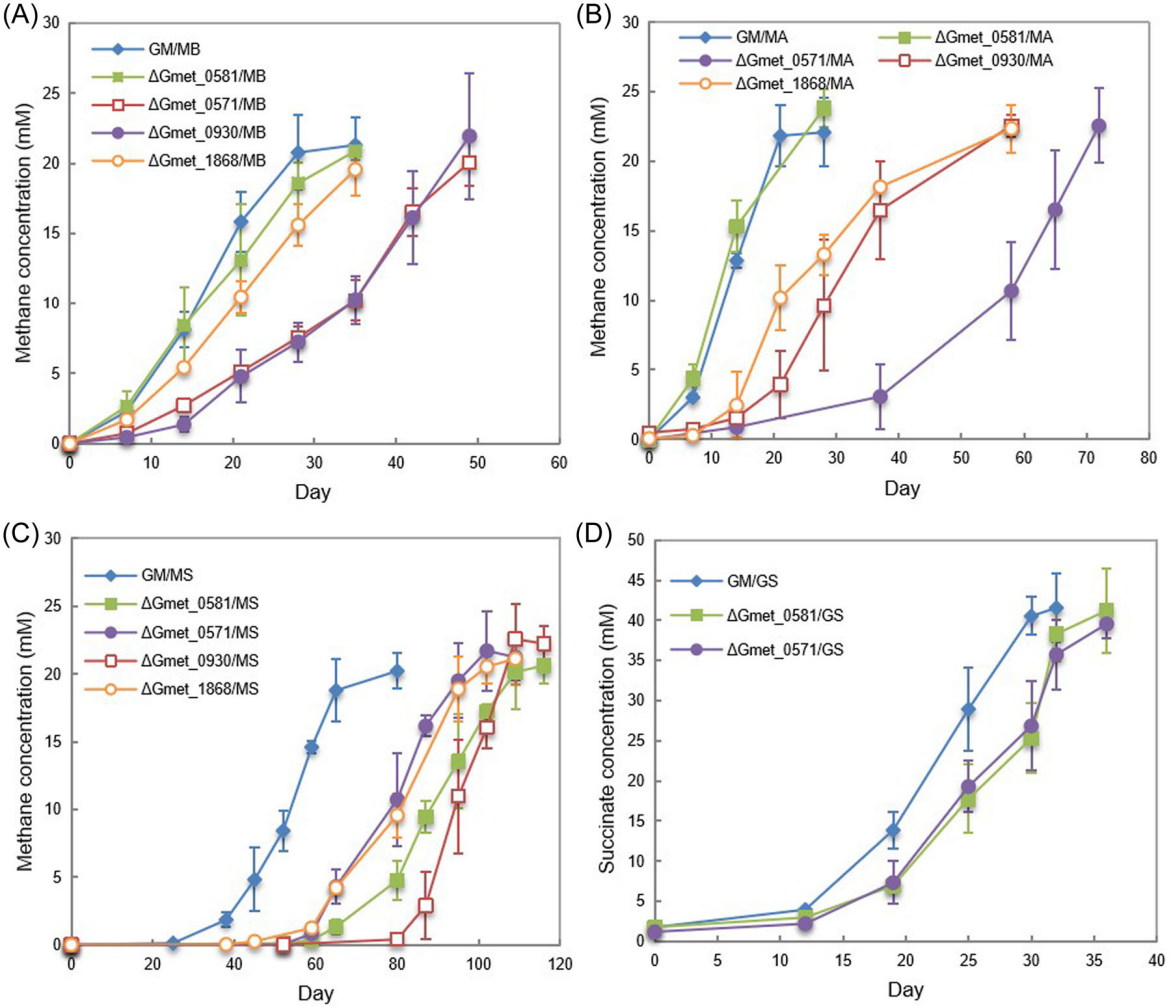
Growth of cocultures with various species of *Methanosarcina* or *Geobacter sulfurreducens* and *G. metallireducens* strains lacking the *c*‐type cytochromes encoded by Gmet_0571, Gmet_0581, Gmet_1868 or Gmet_0930. Methane or succinate production during the first transfer by (A) cocultures with *Methanosarcina barkeri*; (B) cocultures with *M. acetivorans*; (C) cocultures with *M. subterranea*; (D) cocultures with *G. sulfurreducens*. Error bars represent the standard deviation from triplicate cultures.

Gmet_0930 was one of the most highly expressed *G. metallireducens c*‐type cytochrome genes with all electron‐accepting partners (Figure 2 and Table S1). Cocultures could not be established with *G. sulfurreducens* and a strain of *G. metallireducens* in which Gmet_0930 was deleted. Initiating cocultures with the Gmet_0930‐deficient strain delayed methane production by all three of the *Methanosarcina* strains (Figure 4). Although Gmet_0930 was not as essential for DIET with *Methanosarcina* species as it was for DIET with *G. sulfurreducens*, it did appear to optimize DIET because even after four transfers, the *Methanosarcina* cocultures initiated with the Gmet_0930‐deficient strain grew 1.5–2‐fold slower than cocultures initiated with wild‐type *G. metallireducens* (*p* value range, 0.001–0.009; Figure S4). The finding that deletion of Gmet_0930 inhibits DIET with *G. sulfurreducens*, as well as Fe(III) oxide reduction^29^, further supports the conclusion from the study of Gmet_0558, Gmet_0557 and Gmet_2896 that there are differences between *G. metallireducens* electron transport to Fe(III) oxide and *G. sulfurreducens* compared to electron transport to *Methanosarcina* species.

In a similar manner, the four‐heme outer‐surface *c*‐type cytochrome Gmet_1868, which *G. metallireducens* requires for Fe(III) oxide reduction^29^, appeared to be essential for DIET with *G. sulfurreducens*, but only helpful for DIET with the *Methanosarcina* species. Transcript abundance for Gmet_1868 was 4.12 (*p* = 2.39 × 10^−5^) and 2.31 (*p* = 0.001) times greater in GM/GS cocultures than it was in coculture with either of the Type II *Methanosarcina* (GM/MA and GM/MS) (Table S2). Cocultures initiated with a Gmet_1868‐deficient strain and *G. sulfurreducens* did not grow. The lag phase for the Gmet_1868‐deficient strain and *Methanosarcina* cocultures was longer than the wild‐type cocultures (Figure 4) and even after four transfers, the cocultures initiated with the Gmet_1868‐deficient strain and the *Methanosarcina* species grew at rates that were 1.4‐ to 3.3‐fold slower (*p* value range, 0.001–0.03) than the wild‐type coculture (Figure S4).

The 34‐heme outer‐surface *c*‐type cytochrome Gmet_0571, which was highly expressed during growth with all electron‐accepting partners (Figure 2) appeared to be more important for DIET with the *Methanosarcina* species than *G. sulfurreducens*. Cocultures initiated with *G. sulfurreducens* and a strain of *G. metallireducens* in which the Gmet_0571 gene was deleted grew similarly to wild‐type cocultures (Figures 4 and S4). In contrast, cocultures with the Gmet_0571‐deficient strain and *Methanosarcina* were substantially delayed in methane production compared to cocultures initiated with wild‐type *G. metallireducens* (Figure 4). With the subsequent transfer, the ∆Gmet_0571/MB, ∆Gmet_0571/MA and ∆Gmet_0571/MS cocultures adapted somewhat to the loss of Gmet_0571, but they continued to grow 1.6–3.0 times slower (*p* value range, 7.6 × 10^−6^ − 0.04) than the cocultures established with wild‐type *G. metallireducens* (Figure S4). These results suggest that Gmet_0571 is required for optimal DIET with *Methanosarcina* species, but is not necessary for DIET with *G. sulfurreducens* or for growth with Fe(III) oxide provided as the electron acceptor^29^.

A different pattern of gene deletion phenotypes was observed with Gmet_0581, another putative 34‐heme *c*‐type cytochrome likely to be positioned on the outer cell surface. Gmet_0581 shares 63% amino acid identity with the outer membrane *c*‐type cytochrome OmcN of *G. sulfurreducens* and has a surface‐associated immunoglobulin‐like domain. It was highly expressed by all electron‐accepting partners. Deletion of Gmet_0581 impacted coculture establishment with Type II *M. subterranea* but not the other partners. However, after four transfers, cocultures with *M. subterranea* grew at rates that were comparable to cocultures established with wild‐type *G. metallireducens* (Figure S4).

### Porin–cytochrome complexes (Pcc)  are required for DIET

Transcriptomic and gene deletion studies of the Pcc complexes also suggested that there are different requirements for DIET to *Methanosarcina* species than DIET to *G. sulfurreducens*. *G. metallireducens* has genes for three Pcc complexes: PccF (Gmet_0908‐0910), PccG (Gmet_0911‐0913) and PccH (Gmet_0825‐0827). Pcc complexes are thought to be necessary for electron transport across the outer membrane of *Geobacter* species^27^, ^33^. The full suite of genes coding for components of the PccH complex was not highly expressed by *G. metallireducens* in any of the coculture conditions, indicating that PccH was not important for DIET. However, some of the most substantial differences in *G. metallireducens* gene transcript abundance in cocultures with *G. sulfurreducens* versus *M. barkeri* as the electron‐accepting partner were for the genes that code for components of the PccG porin–cytochrome complex (Table 1). The genes from the PccF complex, on the contrary, were more highly expressed by *G*. *metallireducens* grown in the three *Methanosarcina* cocultures (Table 1).

**Figure 5:**
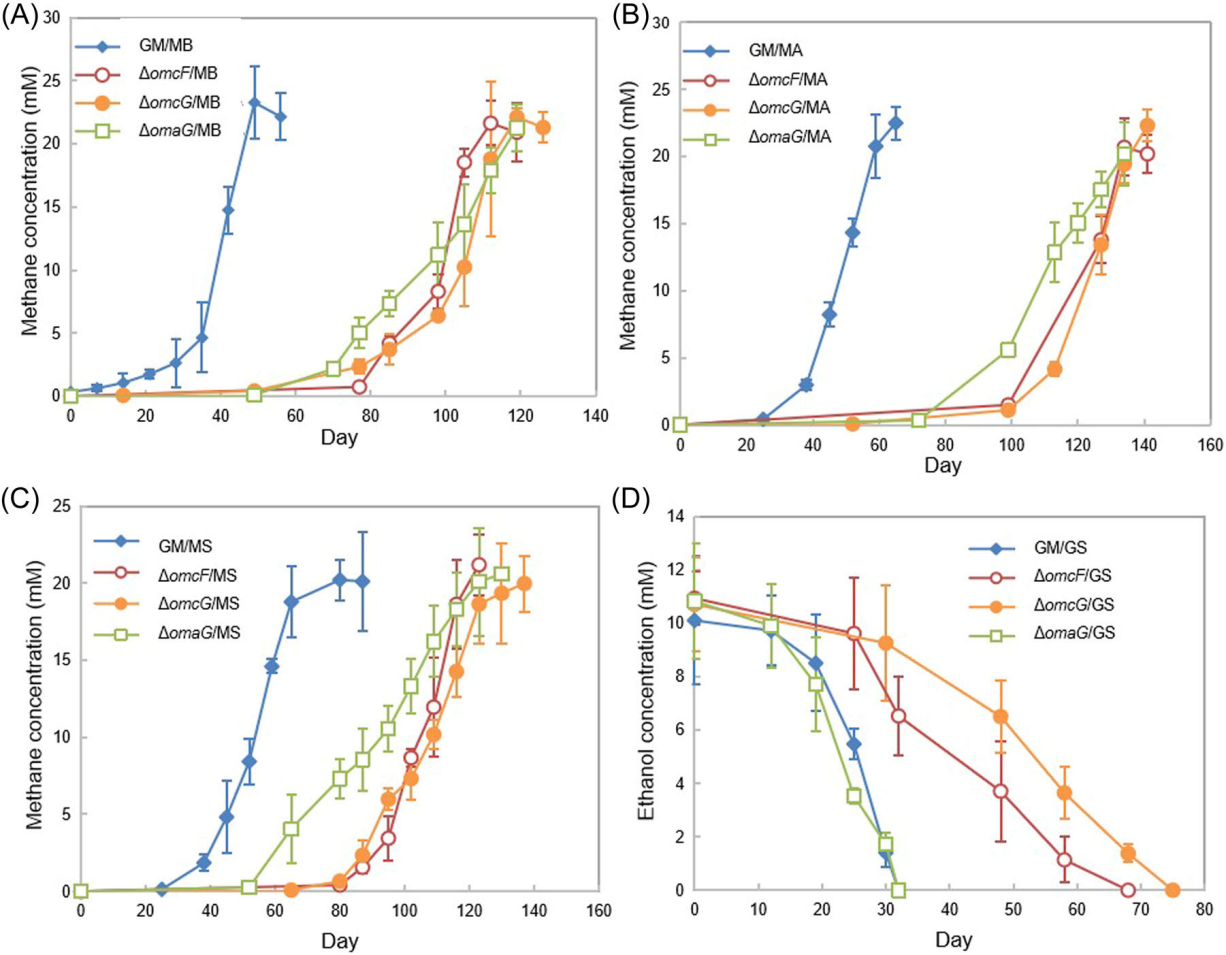
Growth of cocultures with various species of *Methanosarcina* or *Geobacter sulfurreducens* and *G. metallireducens* strains lacking the porin‐associated outer membrane *c*‐type cytochromes OmcF (Gmet_0910), OmcG (Gmet_0913) or the periplasmic porin‐associated cytochrome OmaG (Gmet_0912). Methane production or ethanol consumption during the first transfer by (A) cocultures with *Methanosarcina barkeri*; (B) cocultures with *M. acetivorans*; (C) cocultures with *M. subterranea*; (D) cocultures with *G. sulfurreducens*. Error bars represent the standard deviation from triplicate cultures.

Initiating cocultures with strains of *G. metallireducens* in which the gene for OmcF or OmcG, the outer‐membrane cytochromes of the PccF and PccG conduits, was deleted delayed the initiation of methane production in cocultures established with each of the *Methanosarcina* species (Figure 5A–C). There was also a slight lag in cocultures established with *G. sulfurreducens* and the OmcF*‐* and OmcG*‐*deficient strains of *G. metallireducens* (Figure 5D). Deletion of the gene coding for the periplasmic‐facing *c*‐type cytochrome, *omaG* (Gmet_0912) also delayed methane production with all three *Methanosarcina* species (Figure 5A–C) but did not have an impact on coculture growth with *G. sulfurreducens* (Figure 5D).

After the cocultures were passed through three additional sequential transfers, the cocultures of *G. metallireducens* strain ∆*omcF* and the *Methanosarcina* species, strain ∆*omcF*/*G. sulfurreducens* and strain ∆*omcG*/*G. sulfurreducens*, adapted to grow at rates that were similar to the wild‐type (Figure S5). However, even after this long‐term adaption through multiple transfers, the cocultures that had been initiated with *G. metallireducens* strain ∆*omcG* or strain ∆*omaG* and the *Methanosarcina* species grew ca. 2‐4‐fold slower (*p* value range, 2.6 × 10^−5^ – 0.003) than cocultures initiated with wild‐type *G. metallireducens* (Figure S5). These results suggest that the PccG porin–cytochrome conduit has features that are optimized for DIET with *Methanosarcina* species, but that either porin–cytochrome conduit is suitable for DIET with *G. sulfurreducens*. Strains lacking the gene coding for either of the porin proteins, OmbF or OmbG (Gmet_0908 and Gmet_0911) were not tested for growth in coculture because this study focused on redox proteins required for electron transport out of the cell.

### e‐Pili are important for DIET but are not always required

**Figure 6:**
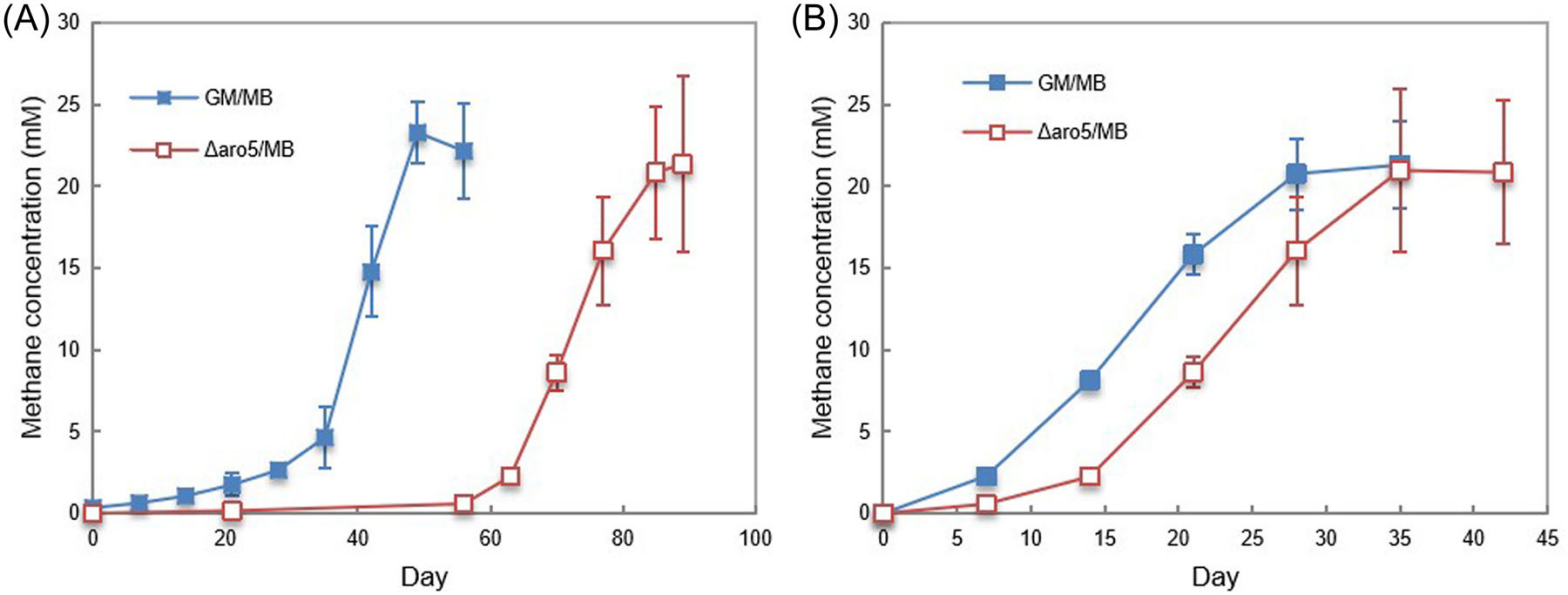
Growth of cocultures with the *Geobacter metallireducens* wild‐type strain or the *G. metallireducens* Aro5 mutant strain and *Methanosarcina barkeri*. Methane production during (A) the first transfer when aggregates became established or (B) the fourth transfer after aggregates had been established. Ethanol (20 mM) was provided as the electron donor. Error bars represent the standard deviation from triplicate cultures.

The *G. metallireducens* gene for PilA, the pilin monomer that is assembled into e‐pili^34^ and the adjacent gene for Spc, a putative chaperone protein that facilitates pilus assembly^35^, as well as many of the pilin accessory proteins^36^, were highly expressed in *G. metallireducens* grown with each of the electron‐accepting partners evaluated (Tables 1 and S4). Constructing *Geobacter* strains that express poorly conductive pili is a method for evaluating the importance of pili conductivity in extracellular electron exchange without disrupting the proper expression of outer‐surface cytochromes that also play a key role in DIET^37^, ^38^, ^39^, ^40^, ^41^. A strain of *G. metallireducens* that expresses poorly conductive pili (strain Aro5) was previously found to be defective in establishing DIET cocultures with *G. sulfurreducens* ^42^ and the Type II *Methanosarcina* species, *M. acetivorans* ^9^. A coculture initiated with *G. metallireducens* expressing poorly conductive pili and the Type I *Methanosarcina* species, *M. barkeri*, was delayed in converting ethanol to methane but adapted to grow in coculture at rates that were similar to the wild‐type cocultures after four transfers (Figure 6). These results suggest that e‐pili are helpful, but not essential for establishing DIET with *M. barkeri*. Eliminating *G. metallireducens* e‐pili expression had no impact on DIET between *G. metallireducens* and *Methanobacteium electrotrophus* strain YSL, which like *M. barkeri* lacks outer‐surface *c*‐type cytochromes^12^. Thus, it appears that e‐pili are more important for establishing electrical contact in species that rely on outer‐surface *c*‐type cytochromes (i.e., *G. sulfurreducens*, *M. acetivorans*) than species that do not (i.e., *M. barkeri*, *M. electrotrophus* strain YSL).

There were also substantial differences in the expression of *G. metallireducens* genes coding for additional surface‐associated proteins, with different electron‐accepting partners (Table S2). In particular, Gmet_2163, coding for a multicopper protein homologous to OmpB, which *G. sulfurreducens* requires for Fe(III) oxide reduction^43^, was more highly expressed in *G. metallireducens/G. sulfurreducens* cocultures than it was in *G. metallireducens* cocultures with the *Methanosarcina* species (Table 1). Further evaluation of the function of the multicopper protein in DIET awaits the construction of the appropriate mutant.

### Failure of *S. oneidensis* to participate in DIET

In addition to illustrating likely differences between routes for DIET from *G. metallireducens* to *G. sulfurreducens* versus *Methanosarcina* species, the results above demonstrate that elucidating the routes for DIET with *G. metallireducens* is complex because of the high abundance of redox‐active proteins on the outer surface, providing opportunities for adapting alternative routes for extracellular electron transfer. A simpler, yet still genetically tractable system to study DIET is desirable^27^. Therefore, the possibility for *S. oneidensis* to serve as the electron‐donating partner in DIET was investigated. Cocultures were initiated with lactate as the electron donor and *S. oneidensis* as the electron‐donating partner and either *M. barkeri*, *M. acetivorans*, *M. horonobensis* or *M. subterranea* as the electron‐accepting partner. None of the cocultures grew or produced methane (data not shown). Effective extracellular electron transfer by *S. oneidensis* is limited to soluble electron acceptors, such as chelated Fe(III), metal ions and electron shuttles, such as flavins^27^, ^44^, ^45^. *S. oneidensis* lacks e‐pili and primarily reduces extracellular particulate electron acceptors, such as Fe(III) oxides and electrodes, with flavins as an electron shuttle^46^, ^47^, ^48^. Although soluble electron shuttles have been shown to facilitate interspecies electron transfer between *Geobacter* species with fumarate serving as the electron acceptor^49^, the redox potential of reduced flavins may be too positive to support the reduction of carbon dioxide by methanogens. The apparent inability of *S. oneidensis* to participate in DIET with methanogens is consistent with the finding that *Shewanella* species are typically not reported to be abundant members of methanogenic communities.

### Implications

The results demonstrate that there may be substantial differences in the electron transport routes for DIET between *G. metallireducens* and *G. sulfurreducens* versus that between *G. metallireducens* and methanogens. Possible reasons for this include variations in key outer‐surface electrical contacts and the difference in the redox potential required for the reduction of fumarate versus carbon dioxide. These distinctions may also account for the observation that all the *G. metallireducens* mutant strains eventually adapted to grow in coculture with *Methanosarcina* species but not with *G*. *sulfurreducens*. Sequencing the genomes of the adapted cocultures might reveal additional mutations associated with these adaptations.

Differences in *G. metallireducens* transcriptomes and the impact of gene deletions on coculture growth suggested that dissimilarities in electron uptake mechanisms between Type I and Type II *Methanosarcina* might also influence *G. metallireducens* physiology during DIET. The results also indicate that the common assumption that extracellular electron transfer to other cells goes through the same pathways as electron transfer to Fe(III) oxide is incorrect, especially for DIET to *Methanosarcina* species. A number of gene deletions previously shown to inhibit *G. metallireducens* growth on Fe(III) oxide had no impact on DIET‐based growth with *Methanosarcina*.

These considerations highlight the need to develop models for DIET with microbes that are relevant to the environment of interest. Although *G. metallireducens/G. sulfurreducens* cocultures with fumarate as the electron acceptor were convenient to develop the concept of DIET^5^, ^6^, ^10^, ^26^, ^50^, ^51^, DIET is most likely to be environmentally significant under methanogenic conditions. Evaluation of *G. metallireducens* growing via DIET with other methanogens, such as *Methanobacterium* ^12^ and *Methanothrix* ^13^ species, which are physiologically and phylogenetically distinct from *Methanosarcina* species are required to more fully understand potential electron transport pathways for DIET. Further analysis of differences in gene expression patterns in diverse electron‐donating microbes and electron‐accepting methanogens will help identify the important electron transport pathways for DIET in methanogenic environments.

The finding that *S. oneidensis* was not capable of DIET with *Methanosarcina* species further demonstrates the often‐ignored substantial differences in the extracellular electron transport capabilities of *Shewanella* and *Geobacter* species. Although *S. oneidensis* is commonly regarded as a model microbe equivalent to *Geobacter* species for the study of anaerobic biogeochemical processes and bioremediation, it lacks many of the metabolic capabilities of *Geobacter* species^27^, ^52^, ^53^. More broadly, these results have implications for the inference for DIET based on metagenomic analyses and suggest that the mere presence of genes for proteins, such as multiheme *c*‐type cytochromes, known to be key components for some forms of extracellular electron transfer, is not necessarily sufficient to confer the capacity for DIET.

## MATERIALS AND METHODS

### Culture media and growth conditions

*Methanosarcina acetivorans* strain WWM1 (∆*hpt*)^54^ was routinely cultured under strict anaerobic conditions at 37°C in MA medium as previously described^9^. *M. barkeri* MS (DSM 800), *M. horonobensis* HB‐1 (DSM 21571) and *M. subterranea* DH‐2^15^ were cultivated in the same medium with 1 g/l NaCl as opposed to the 4 g/l NaCl ordinarily found in the MA medium. All *Methanosarcina* cultures were incubated in an N_2_–CO_2_ atmosphere (80:20, vol/vol) with acetate (40 mM) and methanol (20 mM) provided as substrates for growth.

*Geobacter metallireducens* GS‐15 (ATCC 53774), *G. sulfurreducens* PCA (ATCC 51573) and *S. oneidensis* MR‐1 (ATCC 700550) were routinely cultured at 30°C under anaerobic conditions (N_2_:CO_2_, 80:20, vol/vol). Eighteen different *G. metallireducens c*‐type cytochrome deletion mutant strains constructed as previously described^29^ were obtained from our laboratory culture collection: ∆Gmet_2896, ∆Gmet_0558, ∆Gmet_0534, ∆Gmet_2928, ∆Gmet_2930, ∆Gmet_0557, ∆Gmet_1868, ∆Gmet_0930, ∆Gmet_0679, ∆Gmet_0825, ∆Gmet_0913, ∆Gmet_0910, ∆Gmet_0912, ∆Gmet_0571, ∆Gmet_0232, ∆*fliC*, ∆Gmet_2029 and ∆Gmet_0581. All *G. metallireducens* strains were grown in freshwater medium^55^ with ethanol (20 mM) provided as the electron donor and Fe(III) citrate (56 mM) provided as the electron acceptor. For the growth of *G. sulfurreducens*, acetate (20 mM) was the electron donor and fumarate (40 mM) was the electron acceptor. *S. oneidensis* was grown with lactate (20 mM) provided as the electron donor and Fe(III) citrate (56 mM) provided as the electron acceptor. Growth of *Shewanella* required supplementation of 22 mg/l l‐arginine, 22 mg/l l‐glutamine and 44g/l dl‐serine to the medium.

For coculture experiments with *Methanosarcina* species, *G. metallireducens* strains and either *M. acetivorans*, *M. barkeri* or *M. subterranea* were grown with 20 mM ethanol provided as the electron donor and carbon dioxide as the electron acceptor at 30°C as previously described^9^, ^14^, ^15^. Attempted cocultures with *S. oneidensis* and the various *Methanosarcina* were grown under the same conditions but lactate (20 mM) was provided as the electron donor and the medium was supplemented with the three amino acids mentioned above. For coculture experiments with *Geobacter* species, *G. metallireducens* strains and *G. sulfurreducens* were grown with ethanol (20 mM) provided as the electron donor and fumarate (45 mM) as the electron acceptor in a freshwater medium as previously described^5^. Although *G. sulfurreducens* can disproportionate fumarate^56^, previous studies have demonstrated that fumarate is not disproportionated in *G. metallireducens*/*G. sulfurreducens* DIET cocultures^6^. Furthermore, we monitored ethanol oxidation by the coculture to ensure that growth was not due to fumarate disproportionation. All growth rate *p* values were calculated with an analysis of the variance model using the R Stats package, version 4.0.5^57^.

### Analytical techniques

A gas chromatograph equipped with a headspace sampler and a flame ionization detector (Clarus 600; PerkinElmer Inc.) was used to monitor ethanol concentrations. Methane in the headspace was measured by gas chromatography with a flame ionization detector GC‐8A (SHIMADZU Scientific Instruments Inc.) as previously described^58^ and acetate concentrations were measured with a SHIMADZU high‐performance liquid chromatograph with an Aminex™ HPX‐87H Ion Exclusion column (300mm × 7.8 mm; Bio‐Rad) and an eluent of 8.0 mM sulfuric acid.

### Microscopy

Cells were routinely examined by phase‐contrast and fluorescence microscopy (BV‐2A filter set) with a Nikon E600 microscope (Nikon Instruments Inc.).

### RNA extraction

Cells were harvested from triplicate 50 ml cultures of *G. metallireducens* grown via DIET with *M. barkeri*, *M. acetivorans* or *M. subterranea* during the mid‐exponential phase when ~18 mM methane was detected in the headspace.

Cells were split into 50 ml conical tubes (BD Sciences), mixed with RNA Protect (Qiagen Sciences Inc.) in a 1:1 ratio and pelleted by centrifugation at 3000*g* for 15 min at 4°C. Pellets were then immediately frozen in liquid nitrogen and stored at −80°C. Total RNA was extracted from all samples as previously described^59^ and messenger RNA (mRNA) was further enriched from all samples with the MICROB*Express* kit (Ambion Inc.).

### Illumina sequencing and data analysis

Directional multiplex libraries were constructed with RNA extracted from *G*. *metallireducens/M. barkeri* DIET cocultures (GM/MB), *G. metallireducens/M*. *acetivorans* DIET cocultures (GM/MA) and *G. metallireducens/M. subterranea* DIET cocultures (GM/MS). The ScriptSeq™ v2 RNA‐Seq Library Preparation Kit (Epicentre Technologies) was used to make the libraries according to the manufacturer's instructions and paired‐end sequencing was performed on a Hi‐Seq. 2000 platform at the Deep Sequencing Core Facility at the University of Massachusetts Medical School in Worchester, MA.

Raw data were quality‐checked with FASTQC (http://www.bioinformatics.babraham.ac.uk/projects/fastqc/). All read information from libraries constructed for this study (GM/MB, GM/MA, GM/MS, GM/GS) is provided in Table S5 Trimmomatic^60^ was used to trim and filter all raw paired‐end reads that were then merged with FLASH^61^. Ribosomal RNA (rRNA) reads were removed from the libraries with SortMeRNA^62^.

### Mapping of mRNA reads

Trimmed and filtered mRNA reads from the triplicate samples for the various coculture conditions were mapped against the *G. metallireducens* GS‐15 (NC_007517) genome downloaded from IMG/MER (img.jgi.doe.gov) using ArrayStar software (DNAStar). Reads were normalized and processed for differential expression studies using the edgeR package in Bioconductor^63^. All foldchange and *p* values were obtained using the glmQLFTest function in edgeR, which is a genewise negative binomial generalized linear model that implements the quasi‐likelihood (QL) methods of Lund et al.^64^. All trimmed means of M values were calculated using the calcNormFactors function in edgeR. The plotMDS and plotMD functions in the LIMMA package in RStudio^65^ were used to generate the MDS and MD plots.

## AUTHOR CONTRIBUTIONS

Dawn Holmes and Jinjie Zhou conducted experiments, organized the study and wrote the manuscript. Jessica Smith, Caiqin Wan, and Xinying Liu conducted experiments. Derek Lovley helped to organize the study and to write the manuscript.

## ETHICS STATEMENT

No animal or human research was involved in this study.

## CONFLICT OF INTERESTS

The authors declare no conflict of interests.

## Supporting information

Supporting information.

Supporting information.

Supporting information.

Supporting information.

Supporting information.

## Data Availability

Illumina sequence reads have been submitted to the SRA NCBI database under BioProjects PRJNA727272, PRJNA722959, PRJNA828193 and PRJNA828235 and Biosamples SAMN19011638, SAMN18796025, SAMN27646189 and SAMN27646279.
